# Model-based estimation of AV-nodal refractory period and conduction delay trends from ECG

**DOI:** 10.3389/fphys.2023.1287365

**Published:** 2024-01-12

**Authors:** Mattias Karlsson, Pyotr G. Platonov, Sara R. Ulimoen, Frida Sandberg, Mikael Wallman

**Affiliations:** ^1^ Department of Systems and Data Analysis, Fraunhofer-Chalmers Centre, Gothenburg, Sweden; ^2^ Department of Biomedical Engineering, Lund University, Lund, Sweden; ^3^ Department of Cardiology, Clinical Sciences, Lund University, Lund, Sweden; ^4^ Department of Medical Research, Vestre Viken Hospital Trust, Bærum Hospital, Drammen, Norway

**Keywords:** AV node model, atrial fibrillation, atrioventricular node, mathematical modeling, genetic algorithm, approximate Bayesian computation, ECG, rate control drugs

## Abstract

**Introduction:** Atrial fibrillation (AF) is the most common arrhythmia, associated with significant burdens to patients and the healthcare system. The atrioventricular (AV) node plays a vital role in regulating heart rate during AF by filtering electrical impulses from the atria. However, it is often insufficient in regards to maintaining a healthy heart rate, thus the AV node properties are modified using rate-control drugs. Moreover, treatment selection during permanent AF is currently done empirically. Quantifying individual differences in diurnal and short-term variability of AV-nodal function could aid in personalized treatment selection.

**Methods:** This study presents a novel methodology for estimating the refractory period (RP) and conduction delay (CD) trends, and their uncertainty in the two pathways of the AV node during 24 h using non-invasive data. This was achieved by utilizing a network model together with a problem-specific genetic algorithm and an approximate Bayesian computation algorithm. Diurnal variability in the estimated RP and CD was quantified by the difference between the daytime and nighttime estimates, and short-term variability was quantified by the Kolmogorov-Smirnov distance between adjacent 10-min segments in the 24-h trends. Additionally, the predictive value of the derived parameter trends regarding drug outcome was investigated using several machine learning tools.

**Results:** Holter electrocardiograms from 51 patients with permanent AF during baseline were analyzed, and the predictive power of variations in RP and CD on the resulting heart rate reduction after treatment with four rate control drugs was investigated. Diurnal variability yielded no correlation to treatment outcome, and no prediction of drug outcome was possible using the machine learning tools. However, a correlation between the short-term variability for the RP and CD in the fast pathway and resulting heart rate reduction during treatment with metoprolol (*ρ* = 0.48, *p* < 0.005 in RP, *ρ* = 0.35, *p* < 0.05 in CD) were found.

**Discussion:** The proposed methodology enables non-invasive estimation of the AV node properties during 24 h, which—indicated by the correlation between the short-term variability and heart rate reduction—may have the potential to assist in treatment selection.

## 1 Introduction

Atrial fibrillation (AF) is the most common sustained cardiac arrhythmia and a significant burden for patients and the healthcare system Hindricks et al. (2020). The prevalence of AF is currently estimated to be between 2% and 4% worldwide Benjamin et al. (2019). In addition, the number of AF cases in the European Union is estimated to increase by 89% between 2016 and 2060 Di Carlo et al. (2019). Atrial fibrillation is characterized by disorganized electrical activity in the atria, leading to rapid and irregular contraction, and is associated with an increased risk of mortality, predominantly due to heart failure or stroke Andrew et al. (2013).

The atrioventricular (AV) node acts as the only electrical connection between the atria and ventricles and partly protects the ventricles from the rapid and irregular electrical activity in the atria during AF. It can be functionally divided into two pathways, the fast pathway (FP) and the slow pathway (SP), interconnected at the Bundle of His Kurian et al. (2010). The AV node either blocks an incoming impulse, based on its refractory period (RP), or sends it through with a delay, based on its conduction delay (CD). The AV node is thus the most essential part in regulating the heart rate during AF, and the RP and CD are the two most important properties of the AV node, deciding its filtering capability.

The AV node during permanent AF is in many cases insufficient in regards to maintaining a healthy heart rate. Therefore, the AV node properties are often modified by treatment with rate control drugs, with *β*-blockers and calcium channel blockers recommended as first-line treatment Hindricks et al. (2020). Common *β*-blockers for AF treatment are metoprolol and carvedilol, which block the *β*1 receptors in the heart in order to reduce the effect of the sympathetic nervous system on the heart Dorian (2005). Common calcium channel blockers are verapamil and diltiazem, which prevent the L-type calcium channels in the cardiac myocytes from opening in order to reduce conduction in the AV node Eisenberg et al. (2004). However, due to the significant and poorly understood individual variability, the choice of drug is currently made empirically for each patient Hindricks et al. (2020). This could lead to a prolonged time until successful treatment, and possibly result in a suboptimal final choice of drug. Since the two recommended first-line treatments have different physiological effects on the AV node, assessing the patient-specific properties of the AV node has the potential to assist in treatment selection. Specifically, we hypothesize that *β*-blockers would exhibit an increased effect (more reduced heart rate) when variations in the AV node properties are prominent since *β*-blockers reduce the effect of the sympathetic nervous system.

The AV node has previously been studied using several mathematical models based on invasive data from humans and animals Billette and Nattel (1994); Jørgensen et al. (2002); Mangin et al. (2005); Inada et al. (2009); Climent et al. (2011a); Masè et al. (2012), Masè et al. (2015); Ryzhii and Ryzhii (2023). However, in order for a model to be clinically applicable on an individual level, the model parameters should ideally be identifiable from non-invasive data, such as the electrocardiogram (ECG). A statistical model of the AV node with dual pathway physiology using the RR interval series and the atrial fibrillatory rate (AFR) for model fitting has been proposed Corino et al. (2011), Corino et al. (2013); Henriksson et al. (2015). However, the model lumps RP and CD together, limiting its interpretability.

We have previously proposed a network model of the AV node capable of distinguishing the RP and CD in each pathway Karlsson et al. (2021), together with a framework for continuously estimating its twelve model parameters from 24-h Holter ECG Karlsson et al. (2022). Although promising, the characterization of the AV node was still limited by the number of model parameters and their intrinsic complex dependencies, where a large change in the model parameters could result in a very small change in the RP or CD, thus, making their interpretation a non-trivial task. For a modeling approach to gain acceptance in a clinical context, the outcome should be readily interpretable by medical professionals; a fact that has become especially relevant with the increasing use of advanced modeling and machine learning techniques Teng et al. (2022); Trayanova et al. (2021). Additionally, in Karlsson et al. (2022), a version of Sobol’s method was applied to quantify uncertainty in the parameter estimates. However, these uncertainty estimates were not directly interpretable as probabilities and could thus only be used as a relative measure between the model parameters, between patients, or between different times of the day. When the extent of the uncertainty is unknown, uncertain estimates have the potential to mislead decision-making processes or further analysis of the trends. A proper quantification of the uncertainty is thus advantageous in order to fully understand the estimates.

In the present study, we propose a novel methodology for estimating the RP and CD of both pathways of the AV node and the associated uncertainty continuously over 24 h. The methodology comprises a genetic algorithm (GA) for initial model parameter estimation and an approximate Bayesian computation (ABC) algorithm to refine the estimates, together with a simulation approach to map model parameters to RP and CD in order to increase interpretability. In addition to refining the estimates, the ABC algorithm provides samples from the Bayesian posterior distribution of the AV node properties, hereafter denoted the posterior, enabling proper quantification of the uncertainty of the estimated properties. We use these novel tools in an exploratory manner to analyze Holter ECGs from 51 patients during baseline in combination with their respective drug responses to identify potential markers for differences in drug response. Specifically, we analyze the correlation between diurnal and short-term variability and drug outcomes, as well as train several machine learning models to predict drug outcomes.

## 2 Materials and methods

**FIGURE 1:**
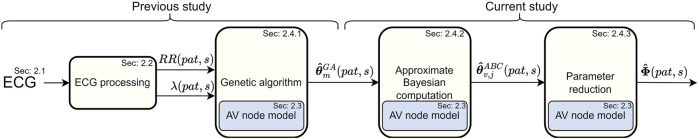
A schematic overview of the methodology, from ECG to estimations of the RP and CD. ${\hat{\theta }}_{m}^{GA}\left(pat,s\right)$ referees to the estimates found by the GA, as described in Section 2.4.1; ${\hat{\theta }}_{v,j}^{\mathrm{ABC}}\left(pat,s\right)$ referees to the estimates found by the ABC algorithm, as described in Section 2.4.2; and $\hat{\Phi }\left(pat,s\right)$ referees to the full estimates of *R* ^*FP*^, *R* ^*SP*^, *D* ^*FP*^, *D* ^*SP*^, as described in Section 2.4.3. Previous study refers to Karlsson et al. (2022).

The overall method for assessing the RP and CD of the two pathways in the AV node for each patient (*pat*) can be divided into four stages, as shown in Figure 1. Firstly, 24-h Holter ECGs are processed to extract RR interval series and AFR trends, divided into 10-min segments (*s*) with a 50% overlap, as described in Sections 2.1, 2.2. Secondly, the parameters for the network model of the AV node, described in Section 2.3, are fitted to the RR interval series and AFR in each segment using a problem-specific dynamic GA as described in Section 2.4.1. The GA-derived estimates are subsequently used as inputs to an ABC algorithm to refine the estimates and estimate the posterior of the model parameters, as described in Section 2.4.2. Additionally, a simulation study was performed to evaluate parameter estimates produced by the ABC algorithm in relation to those produced by the GA, described in Supplementary Material S1. These model parameter estimates are finally used to simulate data with the model while tracking the RP and CD used for the two pathways, as described in Section 2.4.3. This results in a distribution of the RP and CD in the FP and the SP for each 10-min segment. Finally, the possibility to predict treatment outcomes using the estimated distributions is evaluated, as described in Section 2.5.

### 2.1 ECG data

Data from the Rate Control in Atrial Fibrillation (RATAF) study, a randomized, investigator-blind, crossover study, approved by the regional ethics committee and the Norwegian Medicines Agency and conducted in accordance with the Helsinki Declaration, is analyzed in this study Ulimoen et al. (2013). Specifically, 24-h ambulatory ECGs from 60 patients (mean age 71 ± 9 years, 18 women) with permanent AF, no heart failure, or symptomatic ischemic heart disease, recorded during baseline, are used for the estimation of patient-specific AV node properties. In addition to the baseline ECG, the relative change in the 24-h average heart rate (Δ*HR*) for treatment with the two calcium channel blockers verapamil and diltiazem and the two *β*-blockers metoprolol and carvedilol are used to evaluate the therapeutic implications of the estimated AV node properties. The calculation of Δ*HR* is based on the RR interval series extracted from the ECG, as explained in Section 2.2.

### 2.2 ECG processing

The RR interval series is extracted from the ECG for each patient and divided into 10-min segments with a 50% overlap (***RR*** (*pat*, *s*)), where RR intervals following and preceding QRS-complexes with deviating morphology are excluded from the series Lagerholm et al. (2000). Segments with excessive noise can lead to a large number of undetected beats and thus an unrealistically low heart rate. Hence, each 10-min segment is divided into minute-long non-overlapping intervals, and the whole 10-min segment is excluded from further analysis if any 1-min interval has fewer than 20 detected beats. Patients with RR interval series with a total duration shorter than 12 h are excluded from further analysis. The RR interval series corresponding to the four rate control drugs are calculated equivalently.

Spatiotemporal QRST cancellation is employed to extract the f-waves from the ECG Stridh et al. (2001). Subsequently, the fundamental frequency of the extracted f-waves is tracked using a hidden Markov model-based method to extract an AFR trend for each patient with a resolution of 1 minute Sandberg et al. (2008). For time segments where the AFR could not be obtained due to excessive noise, but the RR interval series could, the AFR is set to the closest observed AFR value.

### 2.3 Network model of the AV node

**FIGURE 2:**
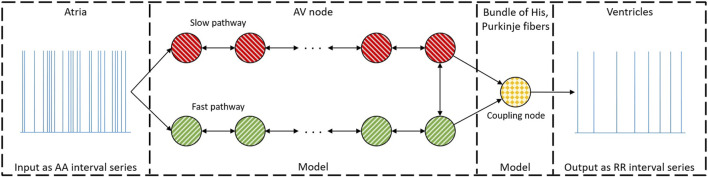
A schematic representation of the network model where the yellow node represents the coupling node, the red nodes the SP, the green nodes the FP, and arrows the direction for impulse conduction. For readability, only a subset of the 21 nodes is shown. Reproduced from Karlsson et al. (2021), licensed under CC BY 4.0.

Our network model of the AV node, introduced in Karlsson et al. (2021), describes the AV node as two pathways (the SP and the FP) comprising 10 nodes each. The last nodes of each pathway are connected with each other and with a coupling node, as illustrated in Figure 2. Each pathway node corresponds physiologically to a localized section of the respective pathway, and the interconnection of the modeled pathways represents the interconnection between the two pathways seen in the AV node Kurian et al. (2010). Furthermore, the coupling node corresponds physiologically to the Purkinje fibers and Bundle of His.

Atrial impulses are modeled by a Poisson process with mean arrival rate *λ*. The impulses are assumed to reach the first nodes of SP and FP simultaneously. Each network node can be either in a refractory state or in a non-refractory state. A node in its refractory state will block incoming impulses, and a node in its non-refractory state will transmit an incoming impulse to all adjacent nodes with an added conduction delay before entering its refractory state. The RP (*R*_*i*_(*n*)) and CD (*D*_*i*_(*n*)) for node *i* are updated for each incoming impulse *n* according to Eqs 1–3,

$$R_{i}\left(n\right)=R_{\min}+\Delta R\left(1-e^{-{\tilde{t}}_{i}\left(n\right)/\tau_{R}}\right)$$ (1)

$$D_{i}\left(n\right)=D_{\min}+\Delta De^{-{\tilde{t}}_{i}\left(n\right)/\tau_{D}},$$ (2)

$${\tilde{t}}_{i}\left(n\right)=t_{i}\left(n\right)-\left(t_{i}\left(n-1\right)+R_{i}\left(n-1\right)\right),$$ (3)

where, ${\tilde{t}}_{i}\left(n\right)$ is the diastolic interval preceding impulse *n* and *t*_*i*_(*n*) is the arrival time of impulse *n* at node *i*. When ${\tilde{t}}_{i}\left(n\right)<0$ , the node is in its refractory state and will block incoming impulses. All parameters are fixed for each pathway, resulting in three model parameters for the RP in the FP $\left(R_{\min}^{FP},\;\Delta R^{FP},\;\tau_{R}^{FP}\right)$ ; three model parameters for the CD in the FP $\left(D_{\min}^{FP},\;\Delta D^{FP},\;\tau_{D}^{FP}\right)$ ; three model parameters for the RP in the SP $\left(R_{\min}^{SP},\;\Delta R^{SP},\;\tau_{R}^{SP}\right)$ ; three model parameters for the CD in the SP $\left(D_{\min}^{SP},\;\Delta D^{SP},\;\tau_{D}^{SP}\right)$ . These twelve model parameters constitute the mode parameter vector ***θ***. In addition, the RP in the coupling node is fixed to the mean of the ten shortest RR intervals in the data, and its CD is fixed at 60 ms Karlsson et al. (2021).

### 2.4 Parameter estimation

For each 10-min segment, the mean arrival rate for the Poisson process *λ* is estimated as the mean of the AFR trend $\left(\hat{\lambda }\left(pat,s\right)\right)$ , and the model parameters $\hat{\theta }\left(pat,s\right)$ are estimated using a GA together with an ABC algorithm.

An error function (*ϵ*) based on the Poincaré plot, i.e., a scatter plot of successive pairs of RR intervals, is used to quantify the difference between ***RR*** (*pat*, *s*) and a simulated RR interval series $\left(\tilde{RR}\right)$ . The successive pairs of RR intervals for ***RR*** (*pat*, *s*) and $\tilde{RR}$ are placed in two-dimensional bins covering the interval between 250 and 1,800 ms in steps of 50 ms, resulting in *K* = 961 bins, which we refer to as the Poincaré histogram. The error function, based on the work presented in Karlsson et al. (2021), is computed according to Eq. 4,

$$\epsilon =\frac{1}{K}\overset{K}{\underset{k=1}{\sum }}\frac{{\left(x_{k}-\frac{1}{t_{\mathrm{norm}}}{\tilde{x}}_{k}\right)}^{2}}{\sqrt{x_{k}}},$$ (4)

where *x*_*k*_ and ${\tilde{x}}_{k}$ are the numbers of RR intervals in the *k*th bin of ***RR*** (*pat*, *s*) and $\tilde{RR}$ , respectively. Additionally, *t*_norm_ acts as a normalizing constant and is calculated as the duration of $\tilde{RR}$ divided by the duration of ***RR*** (*pat*, *s*).

#### 2.4.1 Genetic algorithm

A problem-specific dynamic GA based on the work presented in Karlsson et al. (2022) is used to get an initial estimate of ***θ*** (*pat*, *s*) in every segment. A GA is a metaheuristic, made up of a population of candidate solutions, called individuals in the GA terminology. However, to avoid confusion with individuals in the context of people, here we will call them parameter vectors. Thus, using the problem-specific dynamic GA results in a population of parameter vectors denoted ${\hat{\theta }}_{m}^{GA}\left(pat,s\right)$ , where *m* denotes the *m*th fittest parameter vector in the population after completion of the GA, i.e., the parameter vector with the *m*th lowest *ϵ*. The hyper-parameters in the algorithm are tuned during the optimization using the difference between the Poincaré histograms in pairs of consecutive segments (Δ*P*) Karlsson et al. (2022). This difference is calculated using Eq. 4 with *x*_*k*_ and ${\tilde{x}}_{k}$ as the number of RR intervals in each bin of the current segment and the following one, respectively.

The GA uses a population of 300 parameter vectors, tournament selection, a two-point crossover, and creep mutation. To avoid premature convergence and to increase performance, immigration through replacement of the least-fit parameter vectors in the population is performed, following the work in Karlsson et al. (2022). Furthermore, Δ*P* is used to determine the number of generations that the GA runs before moving to the next data segment, between two and seven. The initialization of the parameter vectors is done using latin hypercube sampling within the ranges given in Table 1. These values also act as boundaries for the model parameters in the GA and are set with guidance from electrophysiological measurements from previous clinical studies while keeping a conservative range to not exclude realistic values. For further details about the algorithm, see Karlsson et al. (2022).

**TABLE 1:** Parameter ranges for the GA and the ABC PMC algorithm.

Parameters	$R_{\min}^{FP},R_{\min}^{SP}$	Δ*R* ^*FP*^, Δ*R* ^*SP*^	$D_{\min}^{FP},D_{\min}^{SP}$	Δ*D* ^*FP*^, Δ*D* ^*SP*^	$\tau_{R}^{FP},\tau_{R}^{SP},\tau_{D}^{FP},\tau_{D}^{SP}$
GA (ms)	[100, 1,000]	[0, 1,000]	[2, 50]	[0, 100]	[25, 500]
ABC (ms)	[30, 1,300]	[0, 1,300]	[0.1, 80]	[0, 130]	[10, 700]

#### 2.4.2 Approximate Bayesian computation

To estimate the posterior $p\left(\theta |RR\left(pat,s\right),\hat{\lambda }\left(pat,s\right)\right)$ , an approximate Bayesian computation population Monte Carlo sampling (ABC PMC) algorithm is used Turner and Van Zandt (2012). The pseudo-code for the problem-specific ABC PMC is shown in Algorithm 1. The ABC PMC uses a set of *N*_*p*_ = 100 particles to estimate the posterior in each RR segment independently, which are updated iteratively for eight iterations (*j*). Each particle corresponds to a model parameter vector, denoted ${\hat{\theta }}_{v,j}^{\mathrm{ABC}}$ , where *v* corresponds to the *v*th particle for the *j*th iteration. Hence, the particles after the eighth iteration are used as the estimate for the posterior. The algorithm is sped up by utilizing the results from the GA to create the initial population. To construct the initial population, twenty particles are drawn from five different normal distributions, having the five most fit parameter vectors in the GA as means, and identical covariance matrices calculated as the covariance of the 25 most fit parameter vectors in the GA. Hence, the five normal distributions are defined as: $N\left({\hat{\theta }}_{1}^{GA}\left(pat,s\right),\Sigma_{\mathrm{init}}\left(pat,s\right)\right)$ , $N\left({\hat{\theta }}_{2}^{GA}\left(pat,s\right),\Sigma_{\mathrm{init}}\left(pat,s\right)\right)$ , $N\left({\hat{\theta }}_{3}^{GA}\left(pat,s\right),\Sigma_{\mathrm{init}}\left(pat,s\right)\right)$ , $N\left({\hat{\theta }}_{4}^{GA}\left(pat,s\right),\Sigma_{\mathrm{init}}\left(pat,s\right)\right)$ , and $N\left({\hat{\theta }}_{5}^{GA}\left(pat,s\right),\Sigma_{\mathrm{init}}\left(pat,s\right)\right)$ , where the covariance matrix **Σ**_*init*_(*pat*, *s*) = Cov $\left({\hat{\theta }}_{1:25}^{GA}\left(pat,s\right)\right)$ where 1 : 25 denotes [1, 2, … , 25] for convenience. During each iteration, each particle has a probability of being chosen based on an assigned weight, computed according to Eq. 5 Beaumont et al. (2009)

$$w_{v,j}={\left(\overset{N_{p}}{\underset{k=1}{\sum }}w_{k,j-1}N\left({\hat{\theta }}_{k,j-1}^{\mathrm{ABC}}|{\hat{\theta }}_{v,j}^{\mathrm{ABC}},\Sigma_{j-1}\right)\right)}^{-1},$$ (5)

where ***w***_*v*,*j*_ is the weight for the *v*th particle in the *j*th iteration and $N\left({\hat{\theta }}_{k,j-1}^{\mathrm{ABC}}|{\hat{\theta }}_{v,j}^{\mathrm{ABC}},\Sigma_{j-1}\right)$ is the probability of ${\hat{\theta }}_{k,j-1}^{\mathrm{ABC}}$ given the normal distribution with mean ${\hat{\theta }}_{v,j}^{\mathrm{ABC}}$ and covariance **Σ**_*j*−1_, where **Σ**_*j*_ = 2Cov $\left({\hat{\theta }}_{1:N_{p},j}^{\mathrm{ABC}}\right)$ . Furthermore, the chosen particle (***θ*** *) is perturbed to create a proposal particle (***θ*** **) using a transition kernel set as $N\left(0,\Sigma_{j}\right)$ Beaumont et al. (2009). The model is used to simulate data using ***θ*** ** to calculate an associated proposal error (ϵ**) according to Eq. 4. If ϵ** is lower than a set threshold (*T*_*j*_), ***θ*** ** is accepted and used in the next iteration of the algorithm; if not, a new particle is chosen and perpetuated to create a new proposal particle. Note that the boundaries for the ABC PMC algorithm are more inclusive compared to the GA to accommodate the full width of the estimated posteriors, as shown in Table 1. A proposal particle outside the boundaries is always rejected. The next iteration starts when *N*_*p*_ new proposal particles have been accepted, and ***w***_*v*,*j*_, *T*_*j*_, and **Σ**_*j*_ are then updated. The threshold changes based on the results from the GA; where $T_{1}={\hat{\theta }}_{10}^{GA}\left(pat,s\right)$ , $T_{2}={\hat{\theta }}_{8}^{GA}\left(pat,s\right)$ , $T_{3}={\hat{\theta }}_{5}^{GA}\left(pat,s\right)$ , $T_{4}={\hat{\theta }}_{3}^{GA}\left(pat,s\right)$ , and $T_{5:8}={\hat{\theta }}_{1}^{GA}\left(pat,s\right)$ . Hence, after the eighth iteration, the *ϵ* for all particles is lower than the *ϵ* for the fittest parameter vectors found by the GA. Thus, the final population is assumed to be *N*_*p*_ samples from $p\left(\theta |RR\left(pat,s\right),\hat{\lambda }\left(pat,s\right)\right)$ .

The hyper-parameters for the ABC PMC algorithm were decided based on empirical tests on simulated data in combination with theoretical indications. The ABC PMC algorithm should ideally be initialized with a particle cloud that is not too compact and not too wide, since both of those alternatives tend to increase the number of iterations until a steady state can be found for the particle cloud. Initial tests on simulated data (not shown) indicated that a good balance was achieved when the initialization was set to drawn samples from five normal distributions with mean values equal to the five fittest parameter vectors found by the GA. Moreover, the stepwise threshold was based on initial tests on simulated data, however, guided by the theoretical indication that the last iteration should yield parameter vectors with an *ϵ* lower than the *ϵ* for the fittest parameter vector found by the GA. The number of iterations was set to eight after simulations indicating that a steady state was reached after eight iterations, as shown in the Supplementary Material S2. Finally, the number of parameter vectors *N*_*p*_ was st to 100 based on available computational resources

Algorithm 1 Calculate $p\left(\theta |RR,\hat{\lambda }\right)$ , given ***RR***, $\hat{\lambda }$ , the model $\tilde{RR}∼$ Model(***θ***, $\hat{\lambda }$ ), the threshold *T*_*j*_, and the initial estimates ${\hat{\theta }}^{GA}$ . The indication (*pat*, *s*) is omitted to avoid redundancy.

At iteration *j* = 1, set the initial population
Set a counter *c* = 1
**for** 1 ≤ *u* ≤ 5 **do**
 **for** $1\leq q\leq \frac{N_{p}}{5}$ **do**
   Set ${\hat{\theta }}_{c,1}^{\mathrm{ABC}}\leftarrow N\left({\hat{\theta }}_{u}^{GA},\Sigma_{\mathrm{init}}\right)$

   Set initial weights $w_{c,1}\leftarrow \frac{1}{N_{p}}$

   Update counter *c* = *c* + 1
 **end for**
**end for**
Set the initial covariance for the transition kernel **Σ**_1_ ← 2Cov $\left({\hat{\theta }}_{1:N_{p},1}^{\mathrm{ABC}}\right)$

At iteration *j* > 1
**for** 2 ≤ *j* ≤ 8 **do**
 **for** 1 ≤ *v* ≤ *N*_*p*_ **do**
  Set ϵ** = inf
  **while** ϵ** > *T*_*j*_ **do**
   Sample one proposal particle from previous iteration $\theta^{*}∼{\hat{\theta }}_{1:N_{p},j-1}^{\mathrm{ABC}}$ with probability $w_{1:N_{p},j-1}$

   Perturb ***θ*** * by sampling $\theta^{**}∼N\left(\theta^{*},\Sigma_{j-1}\right)$

   Simulate data $\tilde{RR}$ from ***θ*** **: $\tilde{RR}∼$ Model $\left(\theta^{**},\hat{\lambda }\right)$

   Calculate ϵ** from Eq. 4 using $\tilde{RR}$ and ***RR***
  **end** **while**
  Set ${\hat{\theta }}_{v,j}^{\mathrm{ABC}}\leftarrow \theta^{**}$

  Update the weight $w_{v,j}\leftarrow {\left(\sum_{k=1}^{N_{p}}w_{k,j-1}P\left({\hat{\theta }}_{k,j-1}^{\mathrm{ABC}}|N\;\left({\hat{\theta }}_{v,j}^{\mathrm{ABC}},\Sigma_{j-1}\right)\right)\right)}^{-1}$ (Eq. 5)
 **end** **for**
  Update the covariance for the transition kernel **Σ**_*j*_ ← 2Cov $\left({\hat{\theta }}_{1:N_{p},j}^{\mathrm{ABC}}\right)$

**end** **for**

#### 2.4.3 Parameter reduction

The posterior estimate of the parameter vector ***θ*** (*pat*, *s*) is obtained using the resulting *N*_*p*_ samples $\left({\hat{\theta }}_{1:N_{p},8}^{\mathrm{ABC}}\left(pat,s\right)\right)$ from the ABC PMC algorithm. Each ${\hat{\theta }}_{1:N_{p},8}^{\mathrm{ABC}}\left(pat,s\right)$ is utilized within the model together with the associated $\hat{\lambda }\left(pat,s\right)$ to simulate a 10-min RR interval series. For each simulation, *R*_*i*_(*n*) and *D*_*i*_(*n*) are stored for each activation *n* in each pathway node *i* and used as the sample distribution of the RP and CD for the SP and the FP, respectively. The samples from these four distributions, denoted as $\hat{\Phi }\left(pat,s\right)=\left[R^{FP}\left(pat,s\right),R^{SP}\left(pat,s\right),D^{FP}\left(pat,s\right),D^{SP}\left(pat,s\right)\right]$ , serves as a translation from the twelve model parameters $\hat{\theta }$ to four more interpretable AV node properties $\hat{\Phi }$ , taking into account not only the model parameters but also the mean AFR associated with the current RR-segment.

To quantify these distributions, their corresponding empirical probability density functions are computed using the MATLAB function ksdensity (MATLAB R2022b) with default bandwidth. From the empirical probability density functions, the maxima are obtained, denoted ${\hat{\phi }}_{\max}\left(pat,s\right)=\left[R_{\max}^{FP}\left(pat,s\right),R_{\max}^{SP}\left(pat,s\right),D_{\max}^{FP}\left(pat,s\right),D_{\max}^{SP}\left(pat,s\right)\right]$ . In addition, the 5th percentile and the 95th percentile are obtained from $\hat{\Phi }\left(pat,s\right)$ , denoted ${\hat{\phi }}_{5}\left(pat,s\right)=\left[R_{5}^{FP}\left(pat,s\right),R_{5}^{SP}\left(pat,s\right),D_{5}^{FP}\left(pat,s\right),D_{5}^{SP}\left(pat,s\right)\right]$ , and ${\hat{\phi }}_{95}\left(pat,s\right)=\left[R_{95}^{FP}\left(pat,s\right),R_{95}^{SP}\left(pat,s\right),D_{95}^{FP}\left(pat,s\right),D_{95}^{SP}\left(pat,s\right)\right]$ , respectively. Furthermore, the number of impulses traveling through the FP and SP (*N*_*FP*_ and *N*_*SP*_, respectively) is stored, and the ratio is denoted as $SP_{\mathrm{ratio}}\left(pat,s\right)=\frac{N_{SP}\left(pat,s\right)}{N_{FP}\left(pat,s\right)+N_{SP}\left(pat,s\right)}$ .

The patient-specific diurnal variability (Δ*DV*) in the AV node properties is quantified by the average value of ${\hat{\phi }}_{\max}$ during daytime (9:00 a.m. to 9:00 p.m.) divided by the average value of ${\hat{\phi }}_{\max}$ during nighttime (2:00 a.m. to 6:00 a.m.). The definitions of day and night are designed to ensure that the patients are awake during the daytime and asleep during the nighttime. In addition, the patient-specific short-term variability in the AV node properties is quantified by the average Kolmogorov-Smirnov distance $\left(\bar{\Delta KS}\right)$ between consecutive segments of $\hat{\Phi }$ during the full 24-h (8:00 a.m. to 8:00 a.m.). The Kolmogorov-Smirnov distance represents the maximal separation between the empirical cumulative distribution functions between consecutive segments Massey Jr (1951).

A significant difference between daytime and nighttime for the average ${\hat{\phi }}_{\max}$ ; the 90% credibility region, quantified by ${\hat{\phi }}_{5}\left(pat,s\right)-{\hat{\phi }}_{95}\left(pat,s\right)$ ; and the average Kolmogorov-Smirnov distance $\bar{\Delta KS}$ is evaluated using the Wilcoxon signed rank test, since all data did not follow a normal distribution according to the Shapiro-Wilk test (*p* < 0.05).

### 2.5 Prediction of treatment outcome

The predictive power of the estimates $\hat{\Phi }$ , ${\hat{\phi }}_{5}$ , ${\hat{\phi }}_{95}$ , ${\hat{\phi }}_{\max}$ , and *SP*_*ratio*_ in relation to Δ*HR* for the different rate control drugs is evaluated in three ways; by analyzing the correlation between the diurnal and short-time variability and Δ*HR*; by training a feature-based regression model on statistical properties of the trends to predict Δ*HR*; and by training a convolutional neural network on the trends to predict Δ*HR*.

To quantify the correlation between diurnal and short-term variability in the AV node properties and Δ*HR* after treatment with the four rate control drugs, Spearman’s rank correlation is used, since the data do not follow a normal distribution according to the Shapiro-Wilk test (*p* < 0.05). Due to the exploratory nature of the study, no hypothesis test is performed and hence no correction of *p*-values is applied Perneger (1998); Althouse (2016).

Three different feature-based regression models (linear regression, random forest Breiman (2001), and k-nearest neighbor Cover and Hart (1967)) are trained on 66 statistical properties of the trends. These statistical properties are: the mean ± std of the four AV node properties ${\hat{\phi }}_{\max}$ during daytime (8 properties), during nighttime (8 properties), and the full 24-h (8 properties); the mean ± std of the 90% credibility region—calculated as the difference between ${\hat{\phi }}_{5}$ and ${\hat{\phi }}_{95}$ —during daytime (8 properties), nighttime (8 properties), and the full 24-h (8 properties); the mean ± std of the *SP*_*ratio*_ during daytime (2 properties), nighttime (2 properties), and the full 24-h (2 properties); Δ*DV* in the four AV node properties (4 properties); the short-term variability in the four AV node properties (4 properties); as well as the age, gender, weight, and height of the patient.

Deep learning approaches have achieved the current state-of-the-art performance for time-series classification and regression Ismail Fawaz et al. (2019). Hence, the prediction of Δ*HR* for the different rate control drug is evaluated using the time series for ${\hat{\phi }}_{5}$ , ${\hat{\phi }}_{95}$ , ${\hat{\phi }}_{\max}$ , *SP*_*ratio*_, AFR, and the RR interval series as an input to three convolutional neural networks with different architectures, based on only fully connected layers Wang et al. (2017), the ResNet architecture Wang et al. (2017), and the Inception architecture Ismail Fawaz et al. (2020), respectively. To incorporate the age, gender, weight, and height of the patients, the last fully connected layer of the networks is modified to also include these properties as input neurons. The networks were trained using the tsai library Oguiza (2022), with the Adam solver Kingma and Ba (2014) and the Huber loss Huber (1992). Leave-one-out cross-validation is used, so that the network is trained on data from all but one patient and tested on the left-out patient. The average mean square error (MSE) of the predicted and true Δ*HR* for the whole population is calculated and compared between approaches.

## 3 Results

As described in Section 2.1, this study is based on a population of 60 patients. However, due to excessive noise, some patients are excluded from analysis, as described in Section 2.2, resulting in a total of 51 patients. The paired significant tests described in Section 2.4.3 are performed on all patients with data for both daytime and nighttime, resulting in a total of 47 patients. In addition, excessive noise in the ECG during treatment with the four rate control drugs leads to missing values for Δ*HR* for some patients. Thus, of the remaining 51 patients at baseline, two lack data for verapamil, three lack data for diltiazem, two lack data for metoprolol, none lack data for carvedilol, and one lacks data for both verapamil and metoprolol. The mean ± standard deviation of Δ*HR* in the population are 19% ± 23% for verapamil; 24% ± 18% for diltiazem, 17% ± 18% for metoprolol; and 11% ± 6% for carvedilol.

The computation of the $\hat{\Phi }\left(pat,s\right)$ is divided into three parts; the GA, the ABC PMC algorithm, and the parameter reduction. All computations were performed on a desktop computer with an AMD Ryzen 9 5900X CPU (using the twelve cores in parallel). Using this setup, the median computation time per patient was 1 h 20 min for the GA, 12 h 30 min for the ABC PMC algorithm, and 6 min for the parameter reduction.

In addition to providing a measure of uncertainty, using the ABC PMC algorithm also reduces *ϵ* compared to only using the GA. This refinement is quantified by the percentual reduction in *ϵ*, calculated as the average $\frac{\epsilon_{1}^{GA}\left(pat,s\right)-\epsilon_{1}^{\mathrm{ABC}}\left(pat,s\right)}{\epsilon_{1}^{GA}\left(pat,s\right)}100$ for each patient and segment, where $\epsilon_{1}^{GA}\left(pat,s\right)$ and $\epsilon_{1}^{\mathrm{ABC}}\left(pat,s\right)$ represent the lowest error value found for the GA and ABC PMC algorithm, respectively. The average refinement ± standard deviation when using the ABC PMC algorithm was 9.14% ± 3.01%. Moreover, a simulation study was performed to validate the proposed model and framework using ground truth data. These results are found in the Supplementary Material S1.

### 3.1 Parameter trends

**FIGURE 3:**
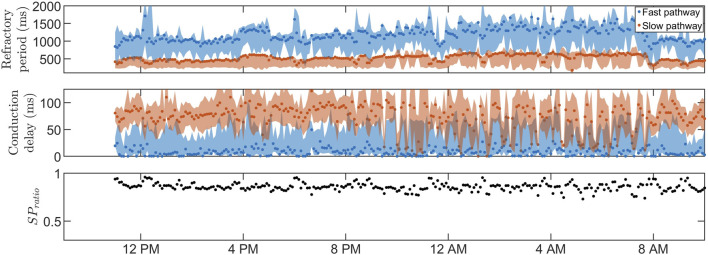
The estimated RP (top) and CD (middle) for ${\hat{\phi }}_{\max}\left(pat,s\right)$ (dotted) as well as ${\hat{\phi }}_{5}\left(pat,s\right)$ and ${\hat{\phi }}_{95}\left(pat,s\right)$ (filled) for the FP (blue) and SP (red), as well as the SP ratio (bottom) are shown for patient A, marked with a black circle in Figure 6.

**FIGURE 4:**
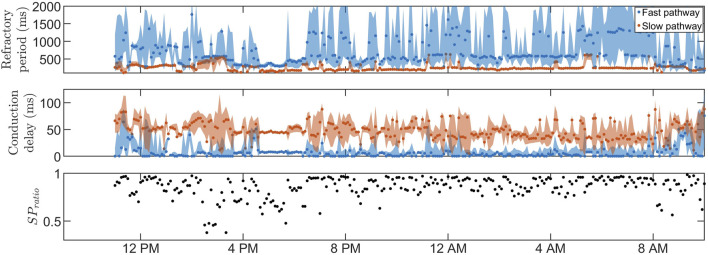
The estimated RP (top) and CD (middle) for ${\hat{\phi }}_{\max}\left(pat,s\right)$ (dotted) as well as ${\hat{\phi }}_{5}\left(pat,s\right)$ and ${\hat{\phi }}_{95}\left(pat,s\right)$ (filled) for the FP (blue) and SP (red), together with the SP ratio (bottom) are shown for patient B, marked with a red circle in Figure 6.

Figures 3, 4 show 24-h trends in estimated RP, CD, and *SP*_*ratio*_ for two patients, denoted patient A (Figure 3) and patient B (Figure 4). Looking at the two top panels of the figures, FP is blue and SP is red. The dots represent the most probable parameter set per segment, ${\hat{\phi }}_{\max}\left(pat,s\right)$ , and colored fields represent the 90% credibility region around the dots, quantified by ${\hat{\phi }}_{5}\left(pat,s\right)$ , and ${\hat{\phi }}_{95}\left(pat,s\right)$ . Comparing the figures, patient A (Figure 3) displays a lower short-term variability, taking values of $\bar{\Delta KS}=\left[0.27,0.19,0.24,0.33\right]$ for *R* ^*FP*^, *R* ^*SP*^, *D* ^*FP*^, and *D* ^*SP*^, respectively. Conversely, patient B (Figure 4) displays a larger variability, with $\bar{\Delta KS}=\left[0.41,0.55,0.40,0.40\right]$ for *R* ^*FP*^, *R* ^*SP*^, *D* ^*FP*^, and *D* ^*SP*^, respectively. Moving on to the bottom panels of Figures 3, 4, it is evident that conduction mainly occurs through the SP in both patients, as indicated by an *SP*_*ratio*_ over 0.5, resulting in a wider credibility region in the *R* ^*FP*^ compared to the *R* ^*SP*^. However, for patient B, there are segments where the FP is more prevalent, e.g., between 5 p.m. and 6 p.m.. In these segments, the RP and CD have a very low variability indicating a stationary behavior of the AV node. A notable shift in RP occurs at 8 a.m. for patient A, probably as a response to waking up from sleep, resulting in a clear change in autonomic regulation. No notable diurnal variability for *R* ^*FP*^, *R* ^*SP*^, and *D* ^*FP*^ could be seen for patient A, with a slight difference in *D* ^*SP*^ (Δ*DV* = [0.80, 0.81, 0.99, 1.39]). For patient B, only *D* ^*FP*^ showed a notable diurnal variability (Δ*DV* = [0.81, 0.92, 2.60, 1.19]).

**FIGURE 5:**
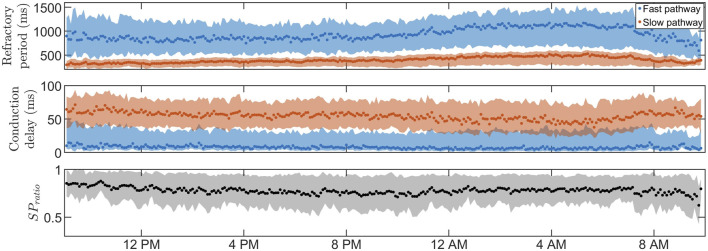
The average RP (top) and CD (middle) for ${\hat{\phi }}_{\max}\left(pat,s\right)$ (dotted) as well as ${\hat{\phi }}_{5}\left(pat,s\right)$ and ${\hat{\phi }}_{95}\left(pat,s\right)$ (filled) for the FP (blue) and SP (red), together with the mean (black, dotted) and standard deviation (black, filled) of the SP ratio (bottom).

Similar observations can be made for the whole population, as displayed in Table 2, which includes the mean and standard deviation of ${\hat{\phi }}_{\max}\left(pat,s\right)$ , the 95% credibility region, and $\bar{\Delta KS}$ , during daytime, nighttime, and during 24 h, as well as Δ*DV*, for the RP and CD in the FP and the SP for all patients. For convenience, the total CD, calculated by multiplying the CD for one node by ten, is listed. Significant difference between daytime and nighttime for ${\hat{\phi }}_{\max}$ , the 90% credibility region, and $\bar{\Delta KS}$ is marked with *, †, and ‡ in Table 2, respectively. From Table 2, it is evident that the RP on average is higher and the CD is lower during nighttime compared to daytime, probably linked to the lower heart rate during sleep and/or circadian autonomic variations. This difference was significant (*p* < 0.001) for *R* ^*FP*^, *R* ^*SP*^, and *D* ^*SP*^, as marked with * in Table 2. Figure 5 illustrates the population average trends of ${\hat{\phi }}_{\max}\left(pat,s\right)$ , ${\hat{\phi }}_{5}\left(pat,s\right)$ , and ${\hat{\phi }}_{95}\left(pat,s\right)$ . To reduce the influence of outliers, only segments containing data from over 20% of the population are shown, resulting in a varying number of patients per plotted segment with a minimum of ten patients per segment and a median of 43 patients per segment. A distinct separation between RP and CD of the two pathways exists, indicating different functionality. Additionally, the credibility region for the *R* ^*FP*^ is larger compared to the *R* ^*SP*^. Moreover, the credibility region for *D* ^*FP*^, in proportion to its mean value, is larger than that of *D* ^*SP*^. The differences in credibility regions between FP and SP reflect the *SP*_*ratio*_, which is 0.78 ± 0.11 (mean ± std) during the day, 0.79 ± 0.12 during the night, and 0.78 ± 0.10 during the full 24-h, indicating that the SP is dominant on average.

**TABLE 2:** The mean ± std of the average ${\hat{\phi }}_{\max}$ , the 95% credibility region, and $\bar{\Delta KS}$ for all patients during daytime, nighttime, and 24-h average together with Δ*DV*. For convenience, the total CD, calculated by multiplying the CD for one node by ten, is listed. A significant difference (*p* <0.001) between the daytime and nighttime estimate is marked by * for ${\hat{\phi }}_{\max}$ , by † for the 90% credibility region ${\bar{\hat{\phi }}}_{95}-{\bar{\hat{\phi }}}_{5}$ , and by ‡ for $\bar{\Delta KS}$ . The indication (*pat*, *s*) is omitted to avoid redundancy.

	*R* ^FP^^* ‡^	*R* ^SP^^*†‡^	10*D* ^*FP*^^‡^	10*D* ^SP^^*†^
24-h ${\bar{\hat{\phi }}}_{\max}$ (ms)	934 ± 203	399 ± 95	76.9 ± 47.6	546 ± 126
Daytime ${\bar{\hat{\phi }}}_{\max}$ (ms)	839 ± 205	356 ± 94	85 ± 64.6	572 ± 139
Nighttime ${\bar{\hat{\phi }}}_{\max}$ (ms)	1,119 ± 294	481 ± 152	62.1 ± 52.8	484 ± 160
24-h ${\bar{\hat{\phi }}}_{95}-{\bar{\hat{\phi }}}_{5}$ (ms)	687 ± 232	217 ± 114	304.1 ± 110.7	447 ± 103
Daytime ${\bar{\hat{\phi }}}_{95}-{\bar{\hat{\phi }}}_{5}$ (ms)	671 ± 261	179 ± 103	299.4 ± 123.9	427 ± 94
Nighttime ${\bar{\hat{\phi }}}_{95}-{\bar{\hat{\phi }}}_{5}$ (ms)	738 ± 290	291 ± 185	315.5 ± 153.3	477 ± 169
24-h $\bar{\Delta KS}$	0.347 ± 0.057	0.319 ± 0.136	0.376 ± 0.055	0.36 ± 0.07
Daytime $\bar{\Delta KS}$	0.368 ± 0.069	0.352 ± 0.169	0.393 ± 0.061	0.351 ± 0.089
Nighttime $\bar{\Delta KS}$	0.309 ± 0.083	0.253 ± 0.133	0.342 ± 0.075	0.38 ± 0.082
Δ*DV*	0.77 ± 0.18	0.78 ± 0.27	2.58 ± 3.72	1.29 ± 0.47

### 3.2 Prediction of treatment outcome

Spearman’s rank correlation between the patient-specific Δ*DV*, as described in Section 2.5, and Δ*HR* showed no clear correlation (*p* < 0.05) for any combination of drug and AV node property. Hence, no relationship between diurnal variability and drug outcome was found.

**FIGURE 6:**
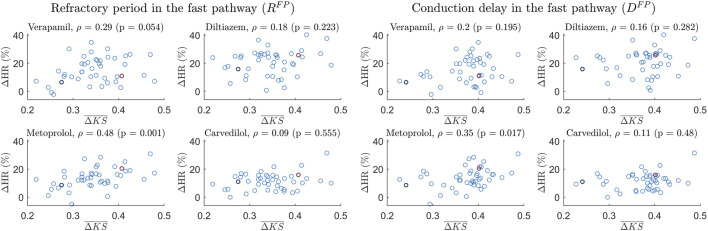
Scatter plot of the 24-h Δ*HR* and $\bar{\Delta KS}$ for the *R* ^*FP*^ (left) and *D* ^*FP*^ (right) for the four drugs with *ρ* indicating the Spearman correlation coefficient, with patient A (as shown in Figure 3) marked with black and patient B (as shown in Figure 4) marked with red.

The Spearman correlation between the patient-specific short-time variability, quantified by $\bar{\Delta KS}$ , and Δ*HR* showed no clear correlation (*p* < 0.05) for the RP and CD in the SP. A moderate correlation was however found between $\bar{\Delta KS}$ and Δ*HR* for *R* ^*FP*^ in the *β*-blocker metoprolol (*ρ* = 0.47, *p* = 0.0011) and for *D* ^*FP*^ in metoprolol (*ρ* = 0.35, *p* = 0.017). Figure 6 shows the individual $\bar{\Delta KS}$ plotted against Δ*HR* and their linear relation for all four drugs, with the left panel showing *R* ^*FP*^ and the right panel showing *D* ^*FP*^. Interestingly, a similar relation between $\bar{\Delta KS}$ , and Δ*HR* is not present in the other *β*-blocker carvedilol.

The ability to predict Δ*HR* using machine learning approaches is evaluated by the average MSE between the predicted and true Δ*HR* for the four drugs using the leave-one-out validation method. The average MSE is benchmarked against the population variance of Δ*HR* for the four drugs. Hence, if the average MSE is larger than the population variance at 0.0071%, the population mean yields a more accurate predictor. Using the feature-based regression models, as described in Section 2.5, resulted in an average MSE of 0.0073% for the linear regression, an average MSE of 0.0074% for the random forest, and an average MSE of 0.074% for the k-nearest neighbor. In addition, using the convolutional neural network resulted in an average MSE of 0.0073% for the fully connected architecture, an average MSE of 0.0079% for the ResNet architecture, and an average MSE of 0.0074% for the Inception architecture. Overall, all the machine-learning approaches resulted in an average MSE higher than 0.0071% and thus in a poor fit to new-seen data.

## 4 Discussion

A mathematical model with an associated framework for patient-specific estimation and proper uncertainty quantification of the RP and CD in the FP and SP of the AV node using only non-invasive data has been proposed.

Individual estimation of trends and variability in AV node properties using non-invasive data has the potential to increase the patient-specific understanding of the AV node during AF, which in turn can be used to enhance informatics approaches for the next-generation of personalized medicine. The two most dominant properties of the AV node, the RP and CD, together with the ratio of impulses conducted through the different pathways, have the potential to increase the understanding of the AV node and its function during AF.

Due to the physiological differences between the effect of *β*-blockers and calcium channel blockers, where *β*-blockers reduce the effect of the sympathetic nervous system, we hypothesized that *β*-blockers could exhibit an increased effect when variations in the AV node properties are prominent since this would indicate a larger influence of the autonomic nervous system. The population-averaged trends (Figure 5; Table 2) show a significant increase in RP for both pathways and a significant decrease in CD for the SP and a non-significant decrease in CD for the FP during nighttime compared to daytime, suggesting that the decreased sympathetic activity during nighttime affects the RP and CD. The PR interval during sinus rhythm can be used as a measure of the CD in the FP for healthy subjects and is known to have a significant increase during nighttime compared to daytime Dilaveris et al. (2001). Interestingly, no corresponding changes in CD for the FP could be observed in our presented analysis, possibly due to the differences in AV node function between AF and sinus rhythm. However, no correlation was found between diurnal variations in AV properties and reduction in heart rate during treatment with *β*-blockers.

Interestingly, a potential association between the short-time variability and the treatment outcome with metoprolol was found. The findings depicted in Figure 6 demonstrate a moderate correlation between $\bar{\Delta KS}$ and the change in heart rate (Δ*HR*) in the RP and CD for the FP for metoprolol, but not for any other drugs or for the SP. The lack of correlation between Δ*HR* after treatment with carvedilol (also a *β*-blocker) and $\bar{\Delta KS}$ could potentially be attributed to its modest overall effect observed in the RATAF study, likely stemming from its rapid elimination as acknowledged in Shapiro (2013). Moreover, the FP and SP are known to have distinct electrophysiological behaviors, hence a different response to drugs between the pathways is to be expected Greener et al. (2011); Nikolaidou et al. (2012); George et al. (2017). For example, the *β*-blocker esmolol has been shown to have a lower effect on the anterograde RP of the SP compared to the FP Philippon et al. (1994). This lower effect on the RP for beta-blockers could possibly explain the lack of correlation seen between the SP estimate and treatment outcome. In general, the mechanisms underlying AV nodal function are debated, and the physiological differences between the pathways that are relevant for the effects of different drug types are not fully known Billette and Tadros (2019). To confirm the association between short-time variability in the RP and CD in the FP and treatment outcome in response to metoprolol, additional studies are needed.

It is possible that predictivity could be improved beyond this association between the short-term variability and the treatment outcome by including additional information from the AV node model. As a tool for this, machine learning techniques are of interest Adam et al. (2020). Hence, three featured-based regression models were used to test if features from the AV node parameter trends could predict Δ*HR* for the different rate control drugs. Moreover, three different architectures of a convolutional neural network were also tested, with the AV node parameter time series as an input, since convolutional neural network have the current state-of-the-art performance for time-series classification and regression Ismail Fawaz et al. (2019). In addition to the estimated AV node parameters, information including the age, gender, weight, and height of the patients was included in an attempt to improve the prediction, since these are immediately available when applying the model in a clinical setting. With a resulting average MSE higher than the variance of Δ*HR* for the population, it appears impossible to predict Δ*HR* with any certainty in the present data set. Either there is not enough information relevant for predicting the heart rate reduction after drug treatment in the AV node property trends—possibly due to the 10-min resolution, limiting the information about autonomic regulation—or the data set size of 51 patients is too low given the inter-individual variability present in the measurements.

Prior iterations of the model and framework focused on estimating the model parameter trends rather than the patient-specific property trends of the AV node Karlsson et al. (2022). This approach imposed limitations on the interpretability of the results, since the interpretation of the model parameters in terms of common cardiology terminology such as RP and CD is not straightforward. In contrast, the current work introduces a novel methodology that enables the estimation of the RP and CD for each ECG segment individually, facilitating a more comprehensible and interpretable analysis. The ability to derive such estimates is vital as it allows for effective communication of the analysis results. Furthermore, this advancement in methodology opens up new avenues for gaining a deeper understanding of the AV node and its diurnal and short-term variations.

The estimation of the posterior by obtaining a range of plausible values, as opposed to relying on a point estimate of the AV node properties, offers notable advantages. For example, the credibility region for *R* ^*FP*^ in Figure 4 is very broad during most segments at nighttime, reflecting a high uncertainty. In scenarios where the extent of the uncertainty is unknown, these uncertain estimates have the potential to influence decision-making processes or further analysis of the trends. As a result, the usefulness and reliability of these estimates may be decreased, emphasizing the need for an estimation of the uncertainty. In our previous work, a GA was used to estimate time variations in the network model parameters during 24 h, with a version of Sobol’s method to quantify the uncertainty in the parameter estimates Karlsson et al. (2022). The uncertainty could be quantified using different methods, such as performing multiple runs of the GA and analyzing the distribution of the resulting estimates or by using bootstrapping to resample the RR interval and run the GA on each resampled dataset. However, the uncertainty estimation resulting from these types of methods, including the version of Sobol’s method previously used, will not be interpretable as probabilities, limiting the reliability of the resulting uncertainty estimates. To produce uncertainty estimates that are interpretable as probabilities, apart from using an ABC approach, the main alternative would be using a Bayesian surrogate model such as the Gaussian process Sudret et al. (2017). However, initial tests found it to be a slower alternative. The ABC approach is well suited for this work since the previously designed error function in Eq. 4 can be used directly as a distance metric, which is often one of the more cumbersome steps in the ABC approach. In addition, the ABC approach has in recent years been used for the personalization of the electrophysiological properties in cardiac models Camps et al. (2021). Although ABC approaches are generally computationally expensive Turner and Van Zandt (2012), starting in a promising area of the model parameter space, derived from the GA results, reduced the computation time by a factor of around 50 (data not shown). The GA was also used to decide on a reasonable threshold level for the ABC PMC algorithm, which is not straightforward since imperfections in the model make certain RR series more challenging to replicate than others, resulting in a higher average *ϵ*. Hence, an *ϵ* value corresponding to a good fit for one RR interval series could correspond to a poor fit for another, making thresholds very data-dependent. Using the GA to find the threshold levels ensures a reasonable threshold level specified for each data segment.

The main advantage of the ABC PMC algorithm is that it provides an estimate of the posterior. Nevertheless, it also has the ability to reduce the *ϵ* value, yielding a closer fit to observed data. The improvement in parameter estimates when combining the GA with the ABC PMC algorithm compared to solely using the GA has been evaluated on simulated data with known model parameters, as shown in the Supplementary Material S1. From this, no statistical difference could be found between the GA and the ABC PMC algorithm for ten out of twelve model parameters when measuring the distance to the known model parameters. However, the best particle found by the ABC PMC algorithm had a significantly lower average *ϵ* value compared to the best parameter vector found by the GA, indicating a better fit to the simulated data. Additionally, in the data analyzed in this study, the best particle for each segment found by the ABC PMC algorithm had on average an *ϵ* value 9.14% lower compared to the best parameter vector found by the GA, confirming an overall improvement.

### 4.1 Study limitations and future perspectives

The AV node model accounts for most properties of importance during AF, such as single and dual pathway physiology, rate-dependent changes in AV conduction properties, and is able to simulate retrograde conduction Billette and Tadros (2019). However, it does not include ventricular escape rhythm, and is unable to replicate the behavior of some rare AV node structures, such as multiple slow pathways. Nevertheless, these simplifications are essential to develop a model with a manageable number of parameters, reducing the computational requirements and thus enabling parameter estimation from non-invasive data using tools such as the GA and the ABC PMC algorithm. Moreover, the model does not explicitly account for AV nodal fatigue. However, any effects of fatigue in the analyzed data sets should be indirectly accounted for in the estimated model parameters.

In this work, we generated the AA interval series used as an input to the model using a Poisson process. We are aware that more detailed representations, notably the Pearson type 4 distribution, can be used to describe atrial impulses during AF Climent et al. (2011b); Plappert et al. (2022). However, for the purposes of the present study, the more simplistic Poisson process was preferred due to its single-parameter description, facilitating parameter estimation, and since it has previously been shown to generate realistic RR-interval series together with the employed AV-node model Karlsson et al. (2021).

The estimated RP and CD have not been validated against intracardiac measurements, since obtaining such measurements during AF—if at all possible—would be very difficult and time-consuming. The average RP and CD for the two pathways can however be compared with invasive electrophysiological measurements of the AV node from two patients with paroxysmal supraventricular tachycardia and evidence of dual AV nodal conduction found in the literature Denes et al. (1973). The two patients had an RP in the FP of 820 ms and 495 ms; an RP in the SP of 540 ms and 414 ms; a CD in the FP of 125 ms and 150 ms; and a CD in the SP of 500 ms and 300 ms. Comparing these values to the daytime estimates seen in Table 2, it is evident that the measured values for the RP and CD in both pathways are within the range of our estimated values. It should be noted that the comparison between AV node properties during paroxysmal and permanent AF is non-trivial, since permanent AF may involve remodeling of the AV node, as shown in animal models Zhang and Mazgalev (2012). Adding to this non-triviality is the fact that the measured functional RP values come from an S1-S2 protocol during sinus rhythm. The functional RP is the smallest AA interval preceding a conducted impulse. It is however still dependent on the previous pacing frequency, which is not well-defined during AF. Nevertheless, since AF leads to high frequencies, the RP should be reasonably close to the functional RP.

In this study, short-time variability was estimated as the difference between adjacent 10-min intervals. Given a constant budget of CPU time, there exists a trade-off between temporal resolution and uncertainty in the estimates, since shorter segments result in an increased number of segments, and more segments result in increased computational demands. Thus, the number of particles would need to decrease, resulting in a poorer estimate of the posterior. Because of this, 10-min segments were chosen to balance the temporal resolution and the quality of the estimates, while keeping the computation time at reasonable levels for practical use. However, the results from the analysis suggest a correlation between short-term variability in the AV node properties and treatment outcome, hinting that increasing the time resolution has the potential to increase the information extracted by the model and framework, which could improve the results. Limiting the short-time variability to 10 minutes also limits the information about the autonomic nervous system—which is known to operate on a higher resolution—to a 10-min resolution. Furthermore, to extract even more information about the impact of the autonomic nervous system on the AV node, an extension of the model has been proposed in Plappert et al. (2022). A similar framework to the one presented in this work could be employed for that model to estimate model parameters and simulate the RP and CD. This could further refine the estimates and thus the information about the AV node.

Moreover, analyzing the RP and CD trends for all the patients, a high inter-individual variability with a wide range of diurnal and short-time variability could be seen, likely due to the inherent individual differences. This, in combination with the relatively low number of patients (51), indicates that the results in this paper should be verified in a larger study.

## 5 Conclusion

We have proposed a novel framework for estimating patient-specific 24-h trends of the RP and CD in the FP and SP of the AV node by mapping estimated model parameters. These estimates include the full posterior of the RP and CD and could be estimated using only non-invasive data. Additionally, a correlation between short-term variability in both the RP and CD for the FP and drug-induced changes to the heart rate was found. The individual estimates of AV node properties offer patient-specific trends in RP and CD, which may have the potential to assist in treatment selection.

## Data Availability

The data analyzed in this study is subject to the following licenses/restrictions: The estimated AV node properties supporting the conclusion for this article will be available from MK upon request. The measured data are owned by Vestre Viken Hospital Trust, and requests for access can be made to SU. The code for the model together with a user example can be found at https://github.com/FraunhoferChalmersCentre/AV-node-model. Requests to access these datasets should be directed to mattias.karlsson@fcc.chalmers.se, sara.ulimoen@gmail.com.
