# Supplementary material for: Model-based estimation of AV-nodal refractory period and conduction delay trends from ECG
Source: Front Physiol. 2024 Jan 12;14:1287365. doi: 10.3389/fphys.2023.1287365 (PMC10811553; doi:10.3389/fphys.2023.1287365)
Supplement: Supplementary file 1 [file DataSheet1.pdf]

Supplementary Material

This supplementary material is divided into two sections. Section 1 contains a simulation study comparing the genetic algorithm (GA) and the approximate Bayesian computation population Monte Carlo sampling (ABC PMC) algorithm. Section 2 covers a simulation study for determining the number of iterations for the ABC PMC algorithm.

1 EVALUATION OF THE GA AND ABC PMC ALGORITHM

The GA and ABC PMC algorithms are evaluated using simulated data generated using 100 parameter vectors ($\boldsymbol{\theta}^{GT}$) with corresponding mean arrival rates (λ) which were randomly selected from the clinical estimates $\hat{\boldsymbol{\theta}}_1^{GA}$ and $\hat{\lambda}$ (cf. Sec 2.4.1). The median and interquartile range of $\boldsymbol{\theta}^{GT}$ and λ are summarized in Table S1. For each parameter vector, a 30-min RR interval series was simulated, denoted $\boldsymbol{RR}^{GT}$.

The GA was applied to estimate $\hat{\boldsymbol{\theta}}_m^{GA}$ from 10-min 50% overlapping segments of $\boldsymbol{RR}^{GT}$, as described in Sec 2.4.1. To avoid transient effects, only the estimates $\hat{\boldsymbol{\theta}}_m^{GA}$ from the last 10-min segment were used for the evaluation. The $\hat{\boldsymbol{\theta}}_m^{GA}$ is used to initiate the ABC PMC algorithm, and the particles from the final iteration are used as an estimate of the posterior, namely $\hat{\boldsymbol{\theta}}_{1:N_p,8}^{ABC}$ (cf. Sec 2.4.2). Furthermore, only the fittest parameter vectors found by the GA and the ABC PMC algorithm, denoted $\hat{\boldsymbol{\theta}}_1^{GA}$ and $\hat{\boldsymbol{\theta}}_1^{ABC}$, are used to evaluate the two algorithms. The median absolute errors (MAE) of $\hat{\boldsymbol{\theta}}_1^{GA}$ and $\hat{\boldsymbol{\theta}}_1^{ABC}$ compared to the ground truth $\boldsymbol{\theta}^{GT}$ are calculated for each of the twelve model parameters, as shown in Table S2. The Wilcoxon signed rank test ($p < 0.05$) is used to evaluate if there is a significant difference between the MAE for the twelve model parameters found by the GA and the ABC PMC algorithm.

To estimate the four AV node properties ($[R^{FP}, R^{SP}, D^{FP}, D^{SP}]$) for the 100 different $\boldsymbol{\theta}^{GT}$, 300 simulations are used (100 using $\hat{\boldsymbol{\theta}}_1^{GA}$, 100 using $\hat{\boldsymbol{\theta}}_1^{ABC}$, and 100 using $\boldsymbol{\theta}^{GT}$). For each of the 300 simulations, $R_i(n)$ and $D_i(n)$ are computed for each activation n in each pathway node i (cf. Sec 2.3) and used as the sample distribution of the refractory period (RP) and conduction delay (CD) for the slow pathway (SP) and the fast pathway (FP), respectively. Note that this is the same procedure used in the main article (Sec 2.4.3) to derive the posterior for the four AV node properties, but used here on only one parameter vector. From this, the mode of the distribution for each of the four AV node properties is obtained for the ground truth, the GA, and the ABC PMC algorithm, denoted $\boldsymbol{\phi}_{max}^{GT}$, $\hat{\boldsymbol{\phi}}_{max}^{GA}$, and $\hat{\boldsymbol{\phi}}_{max}^{ABC}$, respectively. The MAE of $\hat{\boldsymbol{\phi}}_{max}^{GA}$ and $\hat{\boldsymbol{\phi}}_{max}^{ABC}$ compared to the ground truth $\boldsymbol{\phi}_{max}^{GT}$ are calculated for each of the four AV node properties, as shown in Table S3. The Wilcoxon signed rank test ($p < 0.05$) is used to evaluate if there is a significant difference between the MAE for the four AV node properties found by the GA and the ABC PMC algorithm.

The estimates $\hat{\boldsymbol{\theta}}_1^{GA}$ and $\hat{\boldsymbol{\theta}}_1^{ABC}$ as well as the ground truth $\boldsymbol{\theta}^{GT}$ are utilized within the model together with the associated λ to simulate a new RR interval series with a duration of 100 minutes. These new RR interval series are compared with $\boldsymbol{RR}^{GT}$ to compute ϵ (cf. Equation 4, Sec 2.4) for the GA, the ABC PMC algorithm, and the ground truth. Using the $\boldsymbol{\theta}^{GT}$ to simulate a new RR interval series and calculate the ϵ can be viewed as a lower bound for the error. The resulting mean $\pm$ std for the ϵ are shown in Table S4. The Wilcoxon signed rank test ($p < 0.05$) is used to evaluate if there is a significant difference between the ϵ obtained using the GA and the ABC PMC algorithm.

The estimates of R_{min}^{FP} and τ_R^{SP} obtained using the GA have a significantly lower MAE than the

corresponding estimates obtained using ABC PCM ($p = 0.006$ for R_{min}^{FP} and $p = 0.02$ for τ_R^{SP}). However, no significant differences in MAE were found between the estimates in $\boldsymbol{\phi}_{max}^{GA}$ and $\boldsymbol{\phi}_{max}^{ABC}$. Nevertheless, the mean ϵ found by the ABC PMC algorithm is significantly lower compared to the ϵ found by the GA, indicating a better fit to $\boldsymbol{RR}^{GT}$ for the parameter vector found by the ABC PMC algorithm.

Table S1. The median ± interquartile range in ms for the ground truth data.

R_{min}^{FP}	ΔR^{FP}	τ_R^{FP}	R_{min}^{SP}	ΔR^{SP}	τ_R^{SP}	D_{min}^{FP}	ΔD^{FP}	τ_D^{FP}	D_{min}^{SP}	ΔD^{SP}	τ_D^{SP}	λ
549±457	369±441	189±302	217±143	286±378	290±284	4±6	10±34	212±343	35±21	33±50	406±173	6.5±0.6

Table S2. The MAE ± interquartile range in ms for the 12 original model parameters contained in $\hat{\boldsymbol{\theta}}_1^{GA}$ and $\hat{\boldsymbol{\theta}}_1^{ABC}$. Significant differences between the GA and the ABC PMC results are marked with $*$.

	R_{min}^{FP}	ΔR^{FP}	τ_R^{FP}	R_{min}^{SP}	ΔR^{SP}	τ_R^{SP}	D_{min}^{FP}	ΔD^{FP}	τ_D^{FP}	D_{min}^{SP}	ΔD^{SP}	τ_D^{SP}
GA MAE	149±289*	260±282	128±179	18±38	44±79	69±156*	5±9	21±34	129±218	11±14	8±17	115±151
ABC MAE	200±308*	187±287	146±256	21±39	43±72	90±168*	7±10	22±51	170±237	13±15	9±18	116±181

Table S3. The MAE ± interquartile range in ms for the four AV node properties. Significant differences between the GA and the ABC PMC results are marked with $*$ (none were significantly different).

	R^{FP}	R^{SP}	D^{FP}	D^{SP}
GA MAE	152±338	9±29	13±16	12±19
ABC MAE	111±334	12±29	9±18	11±21

Table S4. Mean ± std of the error value for the fittest parameter vector found by the GA and the ABC PMC algorithms are presented, where $*$ indicates a significant difference between the GA and the ABC PMC algorithm, and † indicated a significant difference from the ground truth error.

	GA	ABC	GT
ϵ	483±90*†	456±81*†	420±74

2 DETERMINING THE NUMBER OF ITERATIONS

To decide upon the sufficient number of iterations for the ABC PMC algorithm, an initial simulation study was performed. Too many iterations would increase the computation time, whereas too few iterations would result in ending the algorithm before a steady state for the particle cloud has been found. Five different parameter sets were created, to mimic RR interval series characteristics corresponding to different patients. A 30-minute RR interval series was generated for each simulated patient. Using all impulses traveling through the model for each 30 minute simulation allows quantification of the ground truth distribution of R^{FP}, R^{SP}, D^{FP}, and D^{SP} in terms of the mode, 5th and 95th percentiles, which is used as a lower bound for the estimation accuracy.

The combined GA and ABC PMC algorithm was used to estimate the $\hat{\phi}_{max}$, $\hat{\phi}_5$, and $\hat{\phi}_{95}$, as described in Sec 2.4.1-2.4.3, but using $j = 15$ iterations. In addition, the threshold level (T_j) for $j = 5$ to 15 was set to ϵ for $\hat{\boldsymbol{\theta}}_1^{GA}$. The resulting $\hat{\phi}_{max}$, $\hat{\phi}_5$, and $\hat{\phi}_{95}$ for two of these simulated patients (denoted A and B) are shown in Figures S1 and S2. Note that there is no obvious difference after the eighth iteration. This was true for all five simulated patients, thus j was set to eight. Furthermore, the distance between the $\hat{\phi}_{95}$ and $\hat{\phi}_5$ (blue and red shade in Figures S1 and S2) tends to be wider when the distance between the 5th and 95th percentiles (black shade in Figures S1 and S2) is wide, which is to be expected.

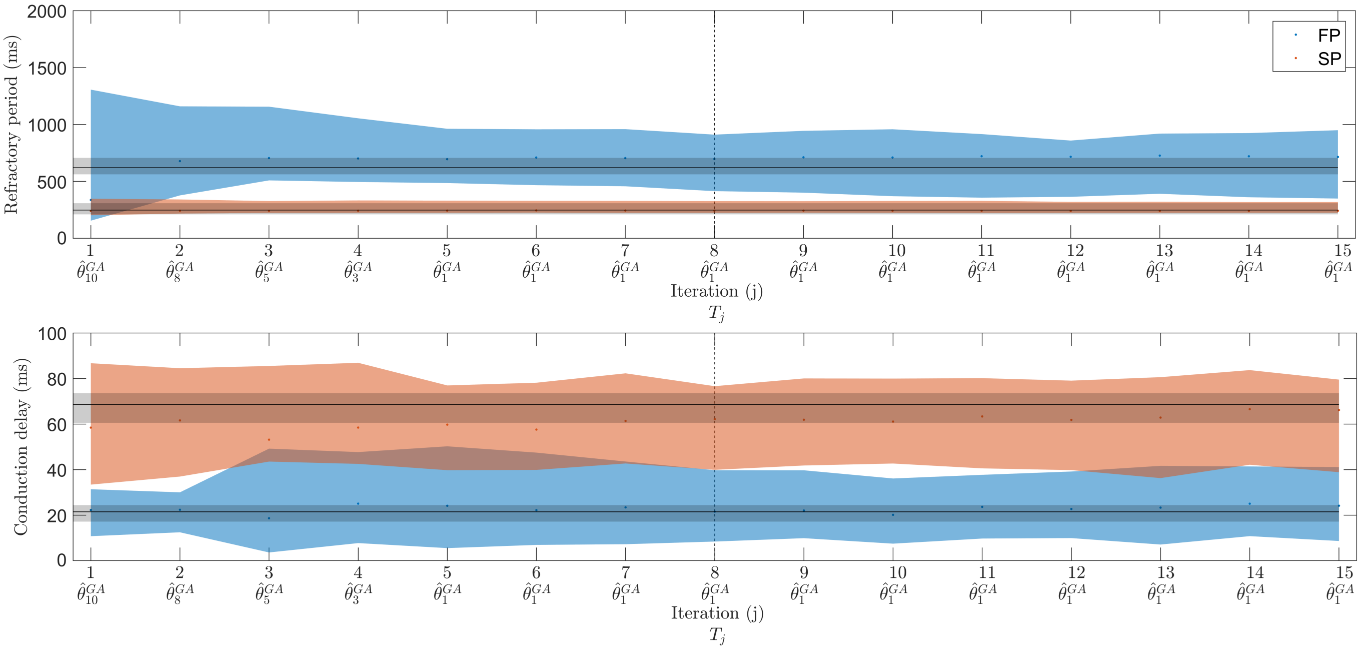

Figure S1. The $\hat{\phi}_{max}$ (dots), $\hat{\phi}_5$ (lower limit of shade), and $\hat{\phi}_{95}$ (upper limit of shade) for the FP (blue) and SP (red) over 15 iterations for patient A. Additionally, the mode (line), the 5th percentile (lower limit of shade), and the 95th percentile (upper limit of shade) for the ground truth (black) are also shown. Moreover, the eighth iteration is marked with a vertical dashed line.

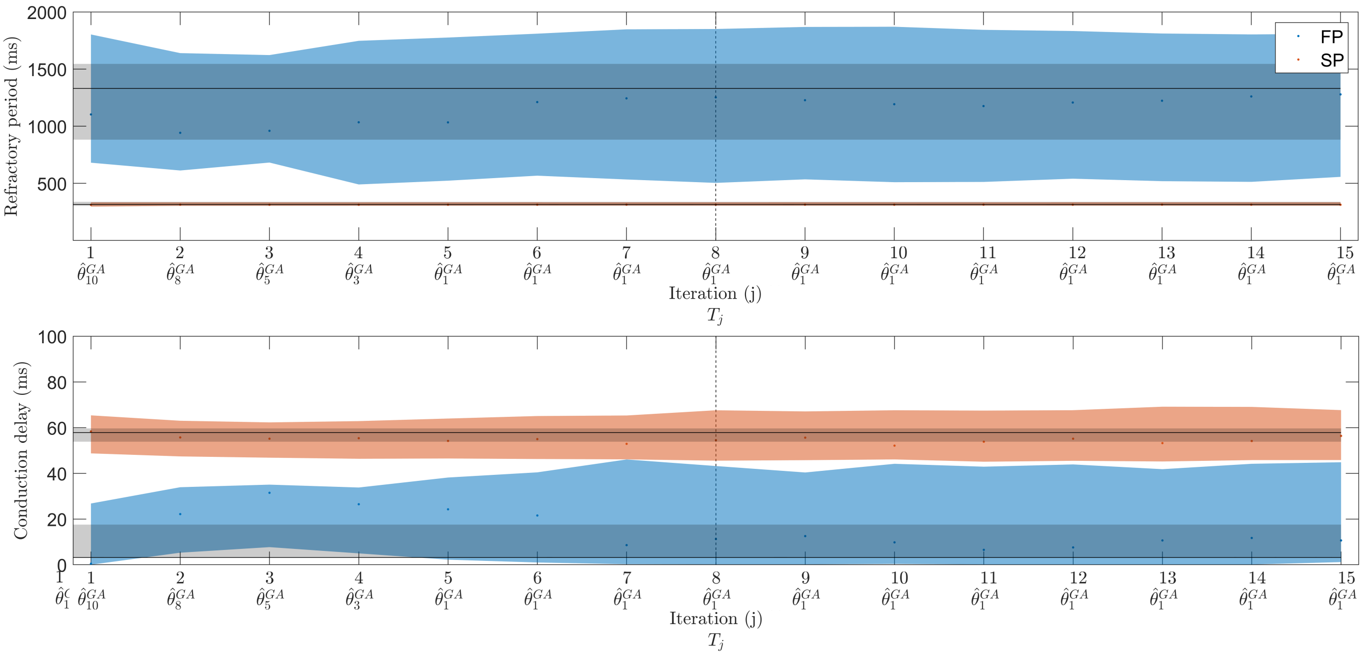

Figure S2. The $\hat{\phi}_{max}$ (dots), $\hat{\phi}_5$ (lower limit of shade), and $\hat{\phi}_{95}$ (upper limit of shade) for the FP (blue) and SP (red) over 15 iterations for patient B. Additionally, the mode (line), the 5th percentile (lower limit of shade), and the 95th percentile (upper limit of shade) for the ground truth (black) are also shown. Moreover, the eighth iteration is marked with a vertical dashed line.
